# Changes in ghrelin, GLP-1, and PYY levels after diet and exercise in obese individuals

**DOI:** 10.1590/1806-9282.20230263

**Published:** 2024-03-15

**Authors:** Gülşah Alyar, Fatma Zuhal Umudum, Nergis Akbaş

**Affiliations:** 1 Atatürk University, Vocational School of Health Services – Erzurum, Turkey.; 2 Atatürk University, Faculty of Medicine, Department of Medical Biochemistry – Erzurum, Turkey.; 3 Yalova University, Faculty of Medicine, Department of Basic Medical Sciences – Erzurum, Turkey.

**Keywords:** Calorie restriction, Exercise, Ghrelin, GLP-1, Obesity, Peptide YY

## Abstract

**OBJECTIVE:**

Diet and exercise, which are the building blocks of obesity management, provide weight loss by creating a negative energy balance. However, the effect of energy deficit induced by long-term diet and exercise on appetite hormones remains unclear. The study was designed to determine the effect of a 12-week diet and exercise program applied to obese individuals on the levels of appetite hormones, namely, ghrelin, GLP-1, and PYY.

**METHODS:**

A total of 62 obese individuals (BMI≥30) and 48 healthy controls (BMI 18.50–29.99) participated in the study. Appropriate diet (1000–1500 kcal/day) and exercise (at least 5000 steps/day) programs were applied to obese individuals according to age, gender, and BMI. The ghrelin, GLP-1, and PYY values of the participants were analyzed by the ELISA method and commercial kit by taking venous blood samples before and after 12 weeks of treatment.

**RESULTS:**

While ghrelin levels of individuals decreased significantly after diet and exercise, PYY levels increased significantly. However, despite the treatment applied, the GLP-1 and PYY levels of the case group did not reach the levels of the control group.

**CONCLUSION:**

Long-term diet and exercise intervention had a positive effect on appetite regulation hormones. It reduced ghrelin levels after treatment. Associated weight loss was facilitated. In the case group, increased satiety hormones after combined treatment supported the maintenance of body weight by increasing satiety.

## INTRODUCTION

The prevalence of overweight and obesity is increasing dramatically around the world. Obesity, which has a complex development with a multifactorial etiology, is mainly caused by positive energy homeostasis^1^. Dietary and exercise interventions, which form the basis of obesity management, reduce body weight by creating a negative energy balance^2^. However, compensatory metabolic and behavioral adaptations in appetite and energy intake in energy deficit may reduce treatment efficacy and lead to weight gain^3^. Acute calorie restriction has been shown to increase hunger and support food intake with an increase in orexigenic signals and a decrease in anorexigenic signals in both obese and normal-weight individuals^4,5^. In contrast, exercise alone can attenuate as well as limit compensatory changes in appetite and energy intake^3^. In fact, it has been determined that the increased energy deficit due to exercise intensity decreases food intake by decreasing hunger hormones and increasing satiety hormones^6^. However, the effect of long-term diet and exercise intervention on appetite hormones in obese individuals remains unclear. Our study was designed to determine the effect of a 12-week diet and exercise applied to obese individuals on ghrelin, GLP-1, and PYY levels. Ghrelin, GLP-1, and PYY are appetite modulators that regulate food intake by mediating neuroendocrine control of energy homeostasis^7^. Ghrelin is a 28-amino acid orexigenic hormone produced extensively by the stomach. As the only stimulator of peripheral food intake, ghrelin has many biological effects, such as stimulating the release of growth hormone and regulating glucose and lipid metabolism^8^. GLP-1 and PYY are anorexigenic hormones secreted from intestinal cells in response to food intake. Satisfaction hormones not only reduce intestinal motility by inducing the "ileal brake" mechanism but also provide appetite and body weight control by acting on the brain stem^9^.

## METHODS

The study, which was approved by the Atatürk University Clinical Research Ethics Committee's decision numbered B.30.2.ATA.0.01.00/31, was carried out in the laboratory of Atatürk University Faculty of Medicine, Department of Medical Biochemistry.

### Materials

The minimum number of samples required for the study was calculated by G Power analysis (version 3.1.9.4) at the level of Type I error (α) 0.05 and Type II error (1–β) 0.85. Accordingly, the t-test in independent groups was determined as at least 24 individuals (Cohen's f: 0.8) for each group, and the t-test in dependent groups was determined as 48 individuals (Cohen's f: 0.8) in each group^10^. The study consisted of individuals who applied to the Erzincan Obesity Center between September 2022 and December 2022. The inclusion criteria for the case group were adult subjects with BMI≥30 kg/m^2^, diabetes mellitus, psychological disorder, and undiagnosed disease that could affect appetite. Exclusion criteria were smoking, psychiatric disorder, and use of any medication with an effect on the appetite mechanism. Inclusion and exclusion criteria for healthy controls consistent with the case group in terms of age and sex were the same, except for having a normal weight (BMI 18.50–29.99). The volunteers were informed about the study, read the "Informed Voluntary Consent Form," and were included in the study after obtaining approval.

### Anthropometric measurements

The BMI of the groups was calculated by dividing the body weight (kg) by the square of the height in meters. Using the BMI classification of the World Health Organization, those with a BMI between 18.50 and 24.99 kg/m^2^ were normal (n=48), and those with a BMI of ≥30 kg/m^2^ were included in the obese (n=62) group.

### Method

In our study, a diet (1000–1500 kcal/day) and physical activity (at least 5000 steps/day) program suitable for age, gender, and BMI parameters was created for the case group. The values of appetite hormones, namely, ghrelin, GLP-1, and PYY in serum samples of the case group were analyzed by the ELISA method and commercial kit by taking venous blood samples before and 12 weeks after starting the diet and physical activity program.

## RESULTS

**Figure 1:**
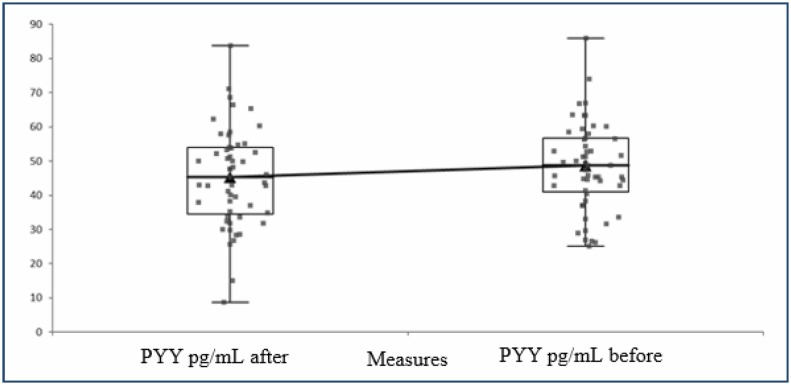
PYY levels before and after 12 weeks of treatment.

**Figure 2:**
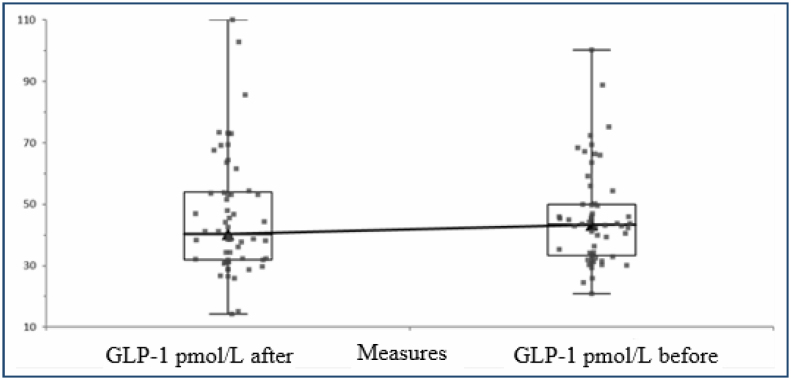
GLP-1 levels before and after 12 weeks of treatment.

**Figure 3:**
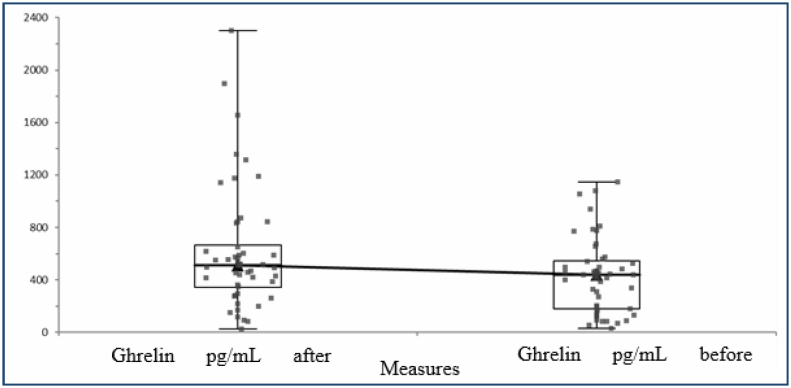
Ghrelin levels before and after 12 weeks of treatment.

A total of 100 individuals with 62 obese and 48 normal body weight were included in the study. PYY values increased significantly after the 12-week treatment program applied to the obese. PYY levels before and after the treatment program are shown in Figure 1. In addition, it was determined that the levels of the hunger hormone ghrelin decreased after the treatment, while the levels of the satiety hormone GLP-1 increased. The ghrelin levels before and after the treatment are shown in Figure 2, and the GLP-1 levels are shown in Figure 3. In addition, post-treatment fasting and satiety hormone levels were found to be lower compared with the control group.

The saturation hormone PYY levels after the treatment program applied to the case group are shown in Figure 1.

The change in the satiety hormone GLP-1 concentration after the treatment program applied to the case group is shown in Figure 2.

The change in ghrelin hormone after the treatment program applied to the case group is shown in Figure 3.

## DISCUSSION

Sustainable weight loss may not always be achieved in the energy deficit created by lifestyle changes (calorie restriction and exercise)^11^. This study evaluates the effect of long-term diet and exercise-induced energy deficit on appetite responses in obese individuals. The appetite-stimulating hormone ghrelin negatively correlates with body weight, while it positively correlates with a low-calorie diet^1^. Similarly, in our study, ghrelin levels of the obese were significantly lower compared with the control group. One of the important findings of our study was that ghrelin levels decreased significantly after the diet and exercise program was applied to obese adults. In parallel with our results, it was determined that ghrelin levels decreased after weight loss with long-term diet and exercise intervention^8^. However, dietary and exercise interventions can initially increase the release of ghrelin triggering an appetite-enhancing response to meet the body's energy needs^12^. The first study on the subject investigated the effects of diet and exercise on food intake and appetite in acute energy deficit induced by diet and exercise in healthy individuals. Accordingly, while the feeling of hunger and food consumption increased in food restriction, perceived hunger and food intake did not change in the energy deficit created by moderate exercise^13^. A study investigating longer (3-day) compensatory responses to diet and exercise in a similar population found that ghrelin levels were similarly elevated. In the same study, PYY3-36 levels were lower in the diet group compared with the exercise group^14^. A study comparing food intake in the equivalent energy deficit induced by diet or exercise in obese adolescents showed an *ad libitum* increase in both acute exercise and dietary restriction. In addition, as a result of the study, a negative correlation was observed between the degree of deficiency induced on the exercise day and energy intake, while a positive correlation was observed with the amount of calories consumed in the *ad libitum* meal on the diet day^15^. In a recent study, the feeling of hunger and food intake in the acute energy deficit created by exercise in adolescents with obesity were higher in the diet group compared with the control and exercise groups^12^. These results suggest that appetite perceptions are sensitive to diet-induced acute energy deficit. However, short-term exercise was not effective in stimulating food intake and appetite, but attenuated compensatory responses. On the contrary, unlike previous studies, our study investigated the effects of a long-term (12-week) diet and exercise intervention applied to obese individuals on both orexigenic and anorexigenic signals. Anotherimportant finding of our study was that PYY levels increased significantly after combined treatment. However, although GLP-1 and PYY levels increased after treatment, they did not reach the levels of those in the control group. The increase in satiety hormone levels was associated with gastrointestinal motility and free fatty acid concentration changes caused by calorie restriction as well as blood redistribution with exercise sympathetic nervous system activity, cytokine release, and lactate production. In a study examining the changes in appetite hormones of intermittent exercise and short-term calorie restriction in obese women, it was determined that fasting PYY levels did not change postprandial PYY increase and desacyl ghrelin decrease. It has been suggested that the energy deficit created by the study treatment program partially affects PYY, strongly suppressing acylated ghrelin^16^. The study that evaluated the effect of long-term fasting and subsequent aerobic exercise on hunger and satiety hormone release found that the level of ghrelin decreased and the secretion of satiety hormone increased^17^. In contrast, Adam et al., studied the effect of a 6-week low-calorie diet on GLP-1 levels in overweight/obese individuals. Contrary to our findings and studies consistent with these results, basal and postprandial GLP-1 levels decreased after weight loss in the study^18^. Accumulating evidence suggests that changes in appetite hormone concentrations depend on the intensity, type, and duration of exercise as well as the study population^2^. Increasing exercise intensity regulates appetite and energy intake by suppressing hunger hormones and stimulating satiety hormones^19^. The study that evaluated the effect of very low volume exercise on appetite hormones in overweight individuals reported that the ghrelin levels of the individuals who exercised were lower than the control, but their GLP-1 levels were higher^20^. The study examining the acute effects of moderate-intensity exercise on appetite hormones in overweight/obese individuals reported that moderate-intensity exercise temporarily inhibited appetite and stimulated PYY and GLP-1 in this population. However, the study found that it did not induce compensatory changes in appetite or energy intake in underweight and overweight/obese subjects^21^. The study that examined the relationship between intense physical exercise and appetite regulation in normal and overweight individuals concluded that PYY was stimulated and ghrelin was suppressed after exercise, and it was suggested that exercise would reduce energy intake^22^. In a study investigating the effect of exercise intensity on appetite and food intake in men with normal body weight, nutrient intake after high-intensity exercise was found to be lower than after moderate-intensity exercise. The ghrelin levels of sedentary controls were found to be higher than those who exercised. As a result, it has been reported that exercise reduces appetite and restricts food intake in this population^19^. In the study investigating the gender-based differences in appetite and energy intake in exercise-induced energy deficit, no statistical difference was observed in ghrelin, PYY, and GLP-1 levels between the sexes after exercise in overweight/obese men and women^23^. In line with all these results, the application of calorie restriction and exercise together helps control weight by having a positive effect on appetite and energy regulation.

## CONCLUSION

The energy deficit induced by long-term diet and exercise positively affected appetite and energy regulation by inhibiting the hunger hormone and stimulating the satiety hormones. Due to the decreased ghrelin levels after the treatment, appetite was suppressed and weight loss was facilitated. After the combined treatment, increased satiety hormones supported the maintenance of body weight by increasing satiety.
